# Multiple myeloma following bone metastasis of renal cell carcinoma: a case report

**DOI:** 10.3389/fendo.2023.1206368

**Published:** 2023-12-01

**Authors:** Hong Yu, Shengnan Zhang, Xiaohui Feng, Feng Gao

**Affiliations:** ^1^ Department of Radiology, The Third Hospital of Hebei Medical University, Shijiazhuang, China; ^2^ Department of Pathology, The Third Hospital of Hebei Medical University, Shijiazhuang, China

**Keywords:** myeloma, bone metastatic tumor, computed tomography, magnetic resonance imaging, diffusion weighted imaging, pathology

## Abstract

**Background:**

The clinical manifestations of multiple myeloma (MM) and bone metastatic tumor are both systemic bone pain, which is difficult to distinguish from imaging manifestations, leading to misdiagnosis and missed diagnosis.

**Case summary:**

We reported a man with a unique case whose tumors were MM with bone metastatic tumor of clear cell renal cell carcinoma (CCRCC). Computed tomography (CT) showed multifocal osteolytic bone destruction, while magnetic resonance imaging (MRI) showed multifocal bone marrow infiltration with soft tissue mass. Pathology and immunohistochemistry established the diagnosis of the coexistence of myeloma with bone metastatic tumor of CCRCC in the spine. Immunotherapy and systemic chemotherapy were adopted in the clinic, and vertebral decompression was performed after anemia was corrected. This case with MM and bone metastatic tumor of CCRCC received radiotherapy and immunotherapy and acquired satisfying outcome after 1 year of follow-up.

**Conclusion:**

It is difficult to differentiate MM and bone metastatic tumor on imaging, especially when there are bone lesions at the same time, which is an easily missed diagnosis and needs to be comprehensively evaluated in combination with functional procedures, clinical laboratory tests, and histopathology.

## 1 Introduction

Multiple myeloma (MM) and bone metastatic tumor are commonly seen malignant tumors in the spine, which are common in the elderly and most present as multiple lesions (1). Their conventional imaging manifestations are similar. Moreover, when the destruction of the vertebral body bone occurs, the bone strength cannot bear the gravity of the human body, from which compression changes can easily develop, that makes the imaging signs of spinal tumors more atypical in nature and further increases the difficulty of diagnosis (2). However, their treatment and prognosis are different. Therefore, how to diagnose correctly is very important.

The patient reported in this case was a 52-year-old man with a unique case, whose tumors in the spine were myeloma following bone metastasis of clear cell renal cell carcinoma (CCRCC). Recently, some studies have postulated an association between MM and renal cell carcinoma (RCC). Only two patients were found to have MM associated with metastatic RCCs (3). We report the first case of both myeloma with bone metastatic tumor of RCC and review the relevant literature.

## 2 Case presentation

This study was conducted under the approval of the ethics committee of the Third Hospital of Hebei Medical University, and the protocol accorded with its standards.

This patient was a 52-year-old man who was admitted to our hospital due to low back pain 2 weeks ago. The symptom of low back pain did not improve after bed rest. He was a chronic smoker for the past 20 years and quit smoking for 5 years. Twenty-nine months previously, he underwent right radical nephrectomy not followed by local radiotherapy. Histopathology of the nephrectomy specimen revealed a CCRCC. No recurrence or distant conversion was found in intermittent reexamination. A routine physical examination revealed a body mass index = 19.27 kg/m^2^ (reference <25) with significant conjuctival pallor but no organomegaly. His blood pressure, pulse rate, respiratory rate, and body temperature were all in the normal range. He had no hypertension, heart disease, diabetes, any infectious disease, or drug allergy history.

**Figure 1:**
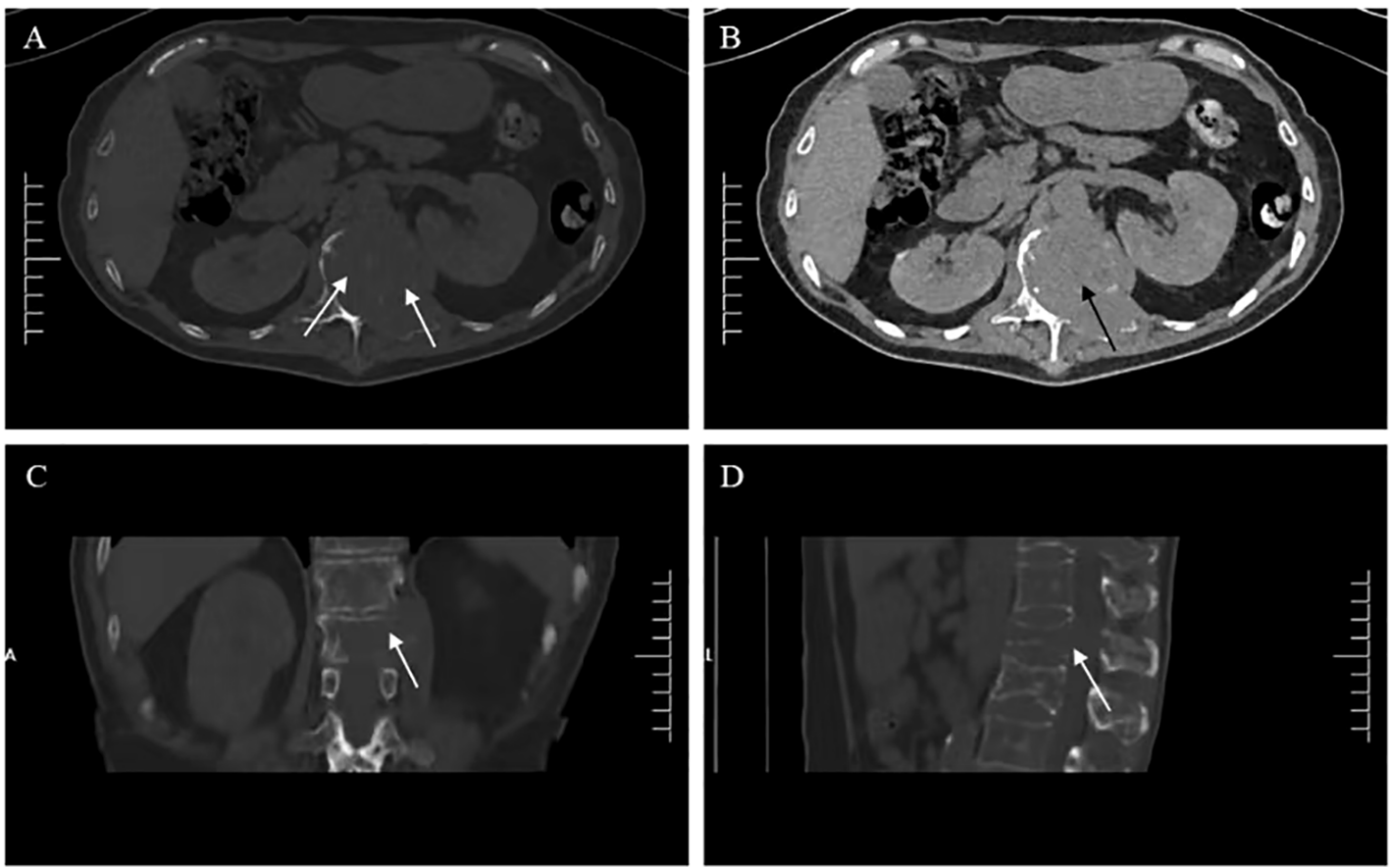
Lumbar computed tomography (CT) showed **(A, C, D)** expansive bone destruction in the vertebrae and appendages of the 11th thoracic vertebra (white arrow), **(B)** accompanied by soft tissue mass formation and vertebral canal compression (black arrow).

**Figure 2:**
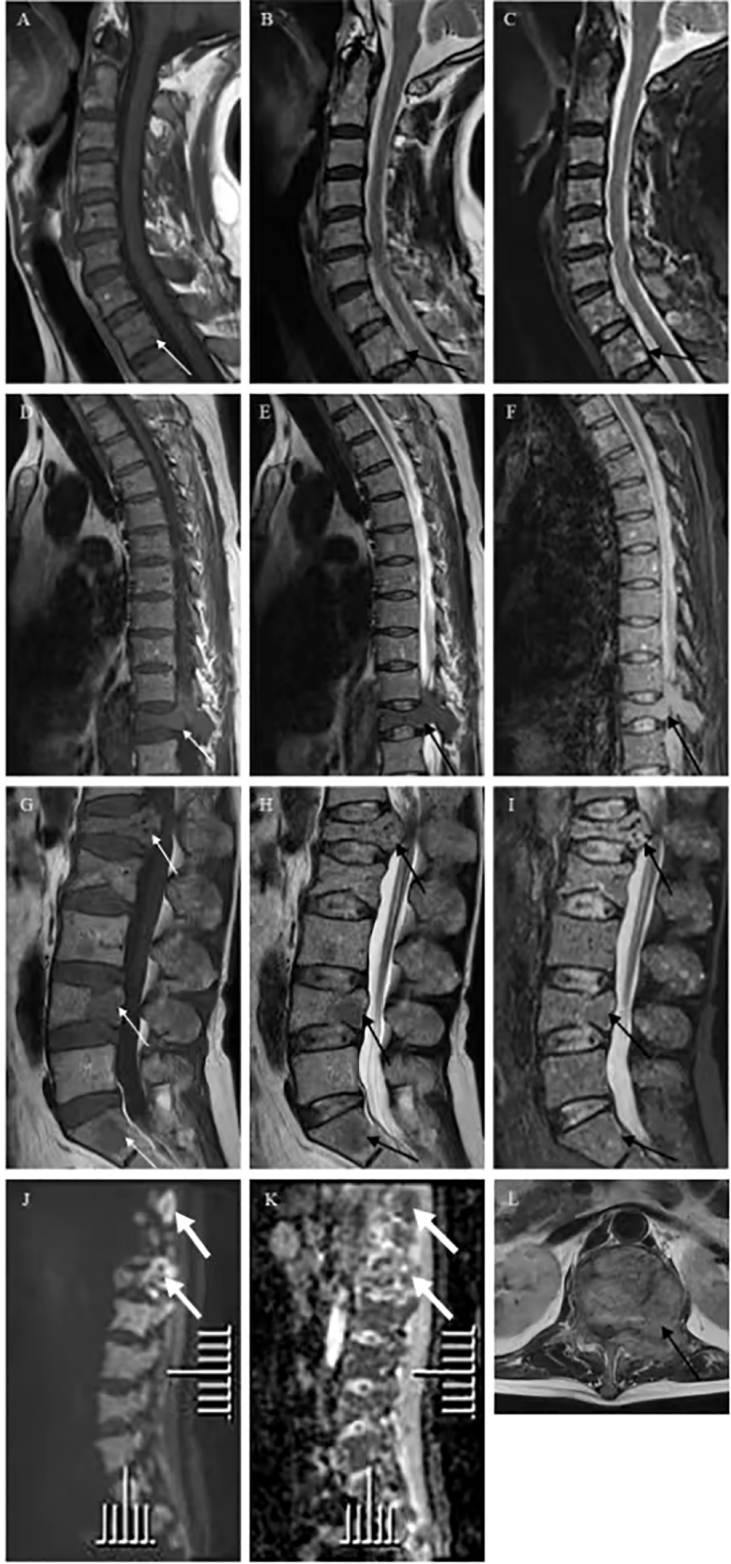
Vertebra magnetic resonance imaging (MRI) and diffusion weighted imaging (DWI) showed multiple spotty abnormal signals in the cervical vertebra, thoracic vertebra, lumbar vertebra, and sacrum, which were both **(A, D, G)** low signal on T1-weighted imaging (white arrow) and **(B, E, H, L)** high signal on T2-weighted imaging and **(C, F, I)** fat suppression sequence (black arrow). DWI of vertebral showed **(J)** scattered high signals, and **(K)** they were low signals on corresponding apparent diffusion coefficient (ADC) imaging (thick white arrow).

Six months ago, he fell down accidentally during work and later developed lumbar pain. He went to the local hospital for computed tomography (CT) examination, which showed lumbar compression fracture, and received local physical therapy. Later, he felt that his symptoms were better than before. However, he had low backache for the last 2 weeks without cause. At day 1 after admission, the lumbar CT revealed expansive bone destruction in the vertebrae and appendages of the 11th thoracic vertebra and the first lumbar vertebra, bone cortex thinning, and local continuity interruption, accompanied by soft tissue mass formation and vertebral canal compression (**Figure 1**). Multiple spotty abnormal signals were seen in the cervical vertebra, thoracic vertebra, lumbar vertebra, and sacrum by magnetic resonance imaging (MRI), which were both low signal on T1-weighted imaging and high signal on T2-weighted imaging, accompanied by mass formation in the 11th thoracic and first lumbar vertebrae and appendages (**Figure 2**). Diffusion weighted imaging (DWI) of vertebral showed scattered high signals, and they were low signals on the corresponding apparent diffusion coefficient (ADC) imaging (**Figure 2**). Pathological fractures were seen on multiple vertebrae suggestive of metastasis of bone or MM.

**Figure 3:**
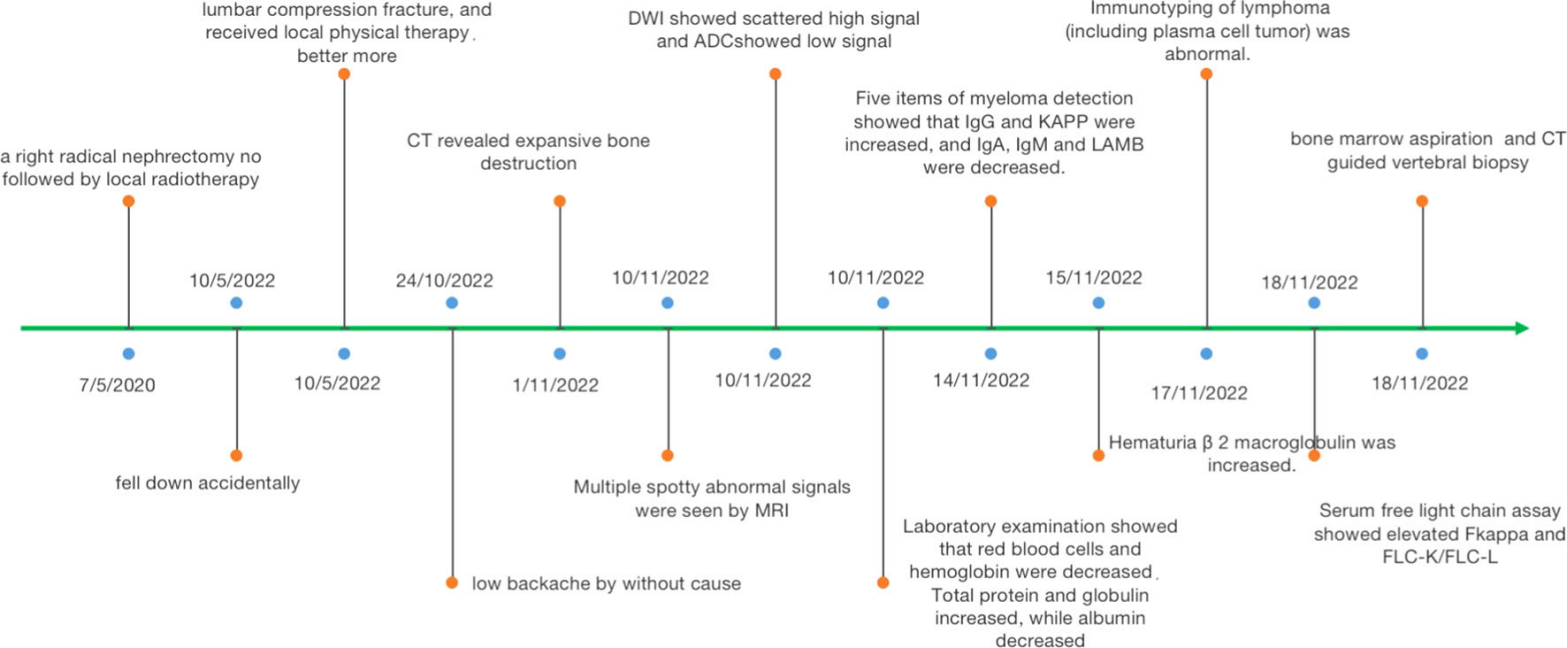
Representative timelines of patient relevant data history.

A routine laboratory examination showed that red blood cells and hemoglobin were decreased. Leukocyte and platelet count were normal. Total protein and globulin increased, while albumin decreased. His serum β 2-microglobulin was 4.59 µg/mL (ref. 0.9–2.7), M protein was 45.79 g/L, and IL-6 was 28.871 pg/mL (ref. <10). Five items of myeloma detection showed that IgG (70 g/L, ref. 7.51–15.6) and immunoglobulin k light chain (KAPPA, 94 g/L, ref. 6.29–13.5) were increased, and immunoglobulin A (IgA; 0.515 g/L, ref. 0.82–4.53), immunoglobulin M (IgM, 0.253 g/L, ref. 0.4–2.74), and immunoglobulin M light chain (LAMBDA, 1.16 g/L, ref. 3.13–7.23) were decreased. Immunotyping of lymphoma (including plasma cell tumor) was abnormal. Serum-free light chain assay showed that elevated serum free kappa was 832.5 mg/L (ref. 3.3–19.4). Blood calcium was reduced, and blood phosphorus was normal. Bone marrow aspiration was MM. Alkaline phosphatase (ALP) was normal (**Figure 3**).

**Figure 4:**
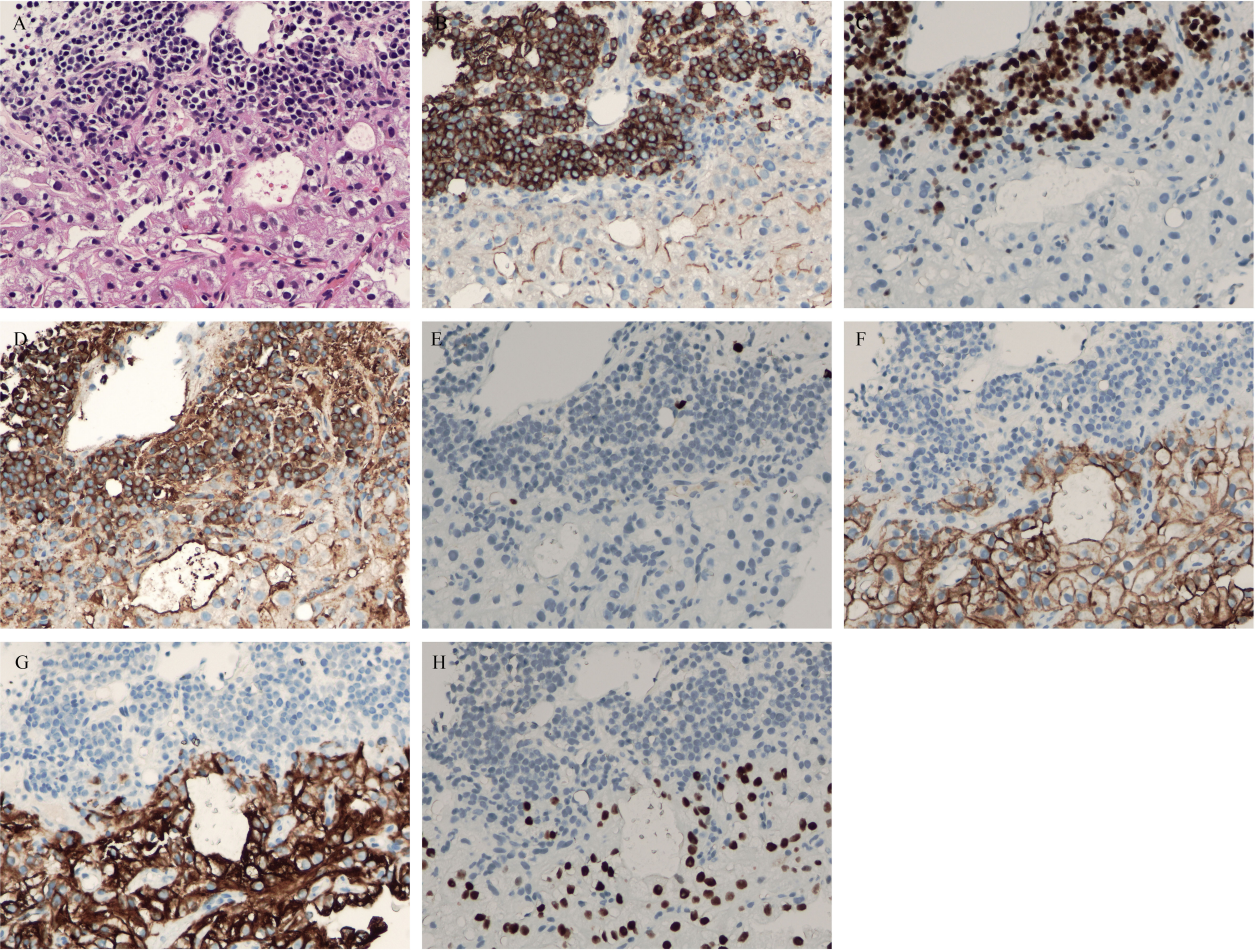
**(A)** hematoxylin and eosin (HE) staining showed coexistence of myeloma with clear cell renal cell carcinoma (CCRCC) in the lumbar spine. **(B)** Positive CD38 immunostaining was observed in myeloma (×200). **(C)** Positive multiple myeloma oncogene1 (MUM1) immunostaining was observed in myeloma (×200). **(D)** Positive kappa immunostaining was observed in myeloma (×200). **(E)** Negative lambda immunostaining was observed in myeloma (×200). **(F)** Positive carbonic anhydrases IX (CA-IX) immunostaining was observed in CCRCC (×200). **(G)** Positive cytokeratin-8 immunostaining was observed in myeloma (×200). **(H)** Positive Pax8 immunostaining was observed in myeloma (×200).

The patient had symptoms of lumbar nerve compression, and surgery was the first choice in clinical practice to relieve the symptoms of nerve compression, but he had anemia. Before surgery, anemia must be corrected and immunotherapy and targeted systemic therapy must be performed. According to the above results, bone marrow aspiration and CT-guided vertebral biopsy were performed to make a definite diagnosis. Pathological examination showed the coexistence of myeloma with CCRCC in hematoxylin and eosin (HE) staining (**Figure 4**). There were monomorphic cells with clear cytoplasm and an intricate network of capillary vasculature in the CCRCC site. The variegated areas of atypical plasma cells infiltrated in CCRCC. There was no clear distinction between the two components. The CCRCC cells were positive for carbonic anhydrase IX (CA-IX), cytokeratin-8, and paired box gene 8 (Pax8); however, the myeloma cells were positive for CD38, CD138, multiple myeloma oncogene 1 (MUM1), and kappa and negative for lambda.

He received radiotherapy and immunotherapy and acquired a satisfying outcome. The patient recovered well after treatment and was followed up for 12 months during the whole treatment course. The symptom of back pain was significantly relieved.

## 3 Discussion

MM and bone metastatic tumor are commonly malignant tumors. The main clinical manifestations of those are the development of osteolytic bone lesions resulting in bone pain and fracture, hypercalcemia, renal insufficiency, and those relating to bone marrow failure (4). MM is an incurable hemopoietic malignancy caused by uncontrolled proliferation of neoplastic plasma cells. CT can detect destructive bone lesions, and MRI can show marrow infiltration (5). Bone metastasis is the most common bone malignant tumor in middle-aged and elderly people, which most common osteolytic metastases are associated with non-Hodgkin lymphoma, non-small cell lung cancer, thyroid cancer, plasmacytoma, Langerhans cell histiocytosis, and RCC (6). Some literature reports showed a bidirectional association between MM and RCC. The relative risk of MM incidence was 51% higher among patients with RCC than in the general population, and the relative risk of RCC incidence was 89% higher among MM patients than in the general population (7).

Myeloma is a hematopoietic malignancy, particularly frequently diagnosed in the sixth to seventh decade of life. Diagnosis of myeloma is based on radiological examinations and detection of monoclonal immunoglobulins or light chains in the urine. MM is localized particularly often in the thoracic and lumbar regions of the spinal column and within its vertebral bodies. The posterior elements are rarely involved. In contrast, bone metastases of spinal are more likely to invade vertebral appendages (8, 9). The imaging manifestation is multiple bone destruction, which can be osteolytic, osteogenic, and mixed. Although MM and bone metastases have some similarities in clinical and imaging, their treatment is quite different. In evaluating surgical treatment, osteosynthesis of a pathological fracture due to bone metastasis is one of the indications for surgical intervention, as is spinal cord involvement or peripheral nerve compression. Surgery can be performed in patients with high risk or imminent pathological fracture. However, MM is mainly treated with chemotherapy (10). Therefore, a clear diagnosis before treatment is essential for treatment.

In recent years, the use of DWI in the clinical setting is becoming more common, which indirectly reflects the abnormal proliferation of tissue cells, and can thus help distinguish different tumors. Literature showed that the ADC value of the bone metastases was higher than those of myeloma in the spine (4). In this study, the ADC value of the appendix of vertebral was higher than that of other parts, and pathology confirmed that metastatic CCRCC and myeloma occurred in the appendix of vertebral together. Combining ADC value with conventional image may markedly aid in the differential diagnosis of metastases and myeloma in the spine. The latest cancer report of the World Health Organization (WHO) stated that approximately 4 million people experienced bone pain due to malignant disease (11). Regardless of the primary malignant, bone metastases are commonly located in the spine, pelvis, shoulder, and distal femur (12).

Pathologically confirmed metastatic CCRCC and myeloma occur together in the spinal appendage. CCRCC should be differentiated from other metastatic cancers, such as lung cancer, gastric cancer, colon cancer, and prostate cancer. Clear cell carcinoma has monomorphic cells with a clear cytoplasm and an intricate network of capillary vasculature. Positive immunohistochemical staining of CA-IX, cytokeratin-8, and Pax8 contributes to the diagnosis of clear cell carcinoma (13, 14). Attention should be paid to the differential diagnosis between myeloma and other small cell tumors. Ewing’s sarcoma is more common in children, while myeloma is more common in the elderly. The typical Ewing’s sarcoma has Homer Wright rosettes formed and does not have the characteristics of plasma cells (15). Myeloma also belongs to a type of lymphoma, which differentiation from other lymphomas is based on immunohistochemical expression in addition to morphology (16). Myeloma is positive for CD38, CD138, and MUM1. Myeloma shows monoclonal expression of kappa or lambda. This patient was positive for kappa and negative for lambda.

This case is rare, which has not been reported before, when multiple focal osteolytic bone destruction occurs with a history of cancer, accompanied by anemia and proteinuria. In this situation, it is necessary to consider both MM and bone metastasis in the diagnosis and provide treatment interventions accordingly.

## Data availability statement

The datasets presented in this study can be found in online repositories. The names of the repository/repositories and accession number(s) can be found in the article/supplementary material.

## Ethics statement

The studies involving humans were approved by the Ethics Committee of the Third Hospital of Hebei Medical University. The studies were conducted in accordance with the local legislation and institutional requirements. The participants provided their written informed consent to participate in this study. Written informed consent was obtained from the individual(s) for the publication of any potentially identifiable images or data included in this article.

## Author contributions

SZ and XF collected the information and images. HY wrote the manuscript. FG reviewed the manuscript. All authors contributed to the article and approved the submitted version.
